# Brg-1 mediates the constitutive and fenretinide-induced expression of SPARC in mammary carcinoma cells via its interaction with transcription factor Sp1

**DOI:** 10.1186/1476-4598-9-210

**Published:** 2010-08-05

**Authors:** Yong Zhong Xu, Mitra Heravi, Thusanth Thuraisingam, Sergio Di Marco, Thierry Muanza, Danuta Radzioch

**Affiliations:** 1 Department of Medicine, Division of Experimental Medicine, McGill University, Montreal, QC, Canada; 2 Department of Human Genetics, McGill University, Montreal, QC, Canada; 3 McGill University Health Centre, Montreal General Hospital Research Institute, Montreal, QC, Canada; 4 Department of Biochemistry, McGill University, Montreal, QC, Canada; 5 Department of Oncology, McGill University, Montreal, QC, Canada

## Abstract

**Background:**

Secreted protein, acidic and rich in cysteine (SPARC) is a matricellular protein that mediates cell-matrix interactions. It has been shown, depending on the type of cancer, to possess either pro- or anti-tumorigenic properties. The transcriptional regulation of the SPARC gene expression has not been fully elucidated and the effects of anti-cancer drugs on this process have not been explored.

**Results:**

In the present study, we demonstrated that chromatin remodeling factor Brg-1 is recruited to the proximal SPARC promoter region (-130/-56) through an interaction with transcription factor Sp1. We identified Brg-1 as a critical regulator for the constitutive expression levels of SPARC mRNA and protein in mammary carcinoma cell lines and for SPARC secretion into culture media. Furthermore, we found that Brg-1 cooperates with Sp1 to enhance SPARC promoter activity. Interestingly, fenretinide [N-4(hydroxyphenyl) retinamide, 4-HPR], a synthetic retinoid with anti-cancer properties, was found to up-regulate the transcription, expression and secretion of SPARC via induction of the Brg-1 in a dose-dependent manner. Finally, our results demonstrated that fenretinide-induced expression of SPARC contributes significantly to a decreased invasion of mammary carcinoma cells.

**Conclusions:**

Overall, our results reveal a novel cooperative role of Brg-1 and Sp1 in mediating the constitutive and fenretinide-induced expression of SPARC, and provide new insights for the understanding of the anti-cancer effects of fenretinide.

## Background

Secreted protein acidic and rich in cysteine (SPARC), also known as osteonectin and BM-40, is a matricellular protein that mediates cell-matrix interaction [1,2]. SPARC plays a role in various physiological processes, including cell adhesion, proliferation, migration, morphogenesis and angiogenesis. It is also involved in processes which require extracellular matrix turnover, such as wound healing and tumor progression [3]. In recent years, the role of SPARC as a modulator in the pathogenesis of different malignancies has become increasingly evident and its role in tumorigenesis appears to be complex, dependent on cell type and tumor microenvironment [4]. SPARC has been shown to function as a tumor suppressor in neuroblastomas, as well as in ovarian, lung, breast, pancreatic and nasopharyngeal cancers[5-15]. Moreover, in tumor xenograft models, the growth of pancreatic and lung cancers in SPARC^-/- ^knockout mice was shown to be significantly enhanced compared with wild-type mice [16,17]. One mechanism proposed for the anti-tumorigenic properties of SPARC is due to its ability to enhance apoptosis [18]. Additionally, the up-regulated expression of SPARC was shown to improve effectiveness of radiotherapy [19] and chemotherapy [20,21] in colorectal cancers. Interestingly, SPARC also has a pro-tumorigenic function linking its expression with poor prognosis in certain human cancers such as melanoma, meningioma and prostate cancer [22-25]. Therefore, more studies are warranted to better delineate the regulation of SPARC and its role in tumor progression.

The modulation of chromatin structure is an essential component in the regulation of both transcriptional activation and repression. Brg-1, one of the ATPase subunits of the SWI/SNF chromatin remodeling complex, plays critical functions in SWI/SNF-mediated transcriptional regulation [26]. It is well established that Brg-1 or Brg-1-containing SWI/SNF complex is involved in either transcriptional activation or transcriptional repression of a subset of genes. For example, Brg-1 is required for the activation of genes such as CD44 [27], MMP-2 [28] and MMP-9 [29], and is required for the repression of genes such as c-fos [30] and cyclin D1 [31]. In addition, Brg-1 has been shown to interact with tumor suppressor p53 [32,33] and β-catenin [34], leading to the transcriptional activation of target genes, as well as tumor suppressor prohibitin [35,36], TopBP [37] and HIC1 [38] mediating transcriptional repression of target genes. As Brg-1 protein does not contain a sequence-specific DNA binding domain, recruitment of Brg-1 or Brg-1-containing SWI/SNF complex to target promoters requires protein-protein interaction between Brg-1 and other transcription factors or transcription regulators. Previous studies have shown that Brg-1 can be recruited to certain gene promoters via its interaction with transcription factor Sp1 [39,40]. Meanwhile, another study demonstrated that Sp1 is bound to the SPARC gene promoter and required for activation of the latter [41]. Taken together, it is not unreasonable to believe that Brg-1 may play an important role in transcriptional regulation of SPARC gene expression.

Fenretinide, a synthetic retinoid with anti-cancer properties, has been widely studied in chemoprevention clinical trials. Prolonged treatment with this drug does not lead to any induction of point mutations or chromosomal aberrations and shows a favorable toxicity profile compared with other classical retinoic acids [42,43]. In rat models of breast cancer, fenretinide selectively accumulates in breast tissue; it is thus particularly active in inhibiting rat mammary carcinogenesis [43,44]. Moreover, in clinical trials, fenretinide decreases the occurrence of secondary breast cancers with a 50% risk reduction in women aged 40 years or younger treated with a low maintenance dose of fenretinide [45]. Furthermore, fenretinide inhibits cell growth through the induction of apoptosis rather than differentiation [46,47], an effect that is strikingly different from that of the parental compound all-trans retinoic acid; it shows synergistic response with chemotherapeutic drugs such as cisplatin, carboplatin, etoposide or TRAIL/Apo2L [48-50]. All of these properties make fenretinide an attractive candidate for cancer chemoprevention and chemotherapy [47,51]. However, the molecular mechanism responsible for these divergent functions of the fenretinide has not yet been fully defined and deserves further investigation.

In this study, we identified Brg-1 as a critical regulator for the constitutive expression of the SPARC gene in mammary carcinoma cell lines. We described, for the first time, the functional importance of the interaction between Brg-1 and Sp1 when binding to the SPARC promoter. We also reported that fenretinide up-regulates the SPARC gene expression via induction of Brg-1. Finally, our results demonstrated that modulation of SPARC is linked to metastatic cancer cell invasion. Overall, our results reveal a novel regulatory mechanism mediating the expression of SPARC and provide new insights for the understanding of the anti-cancer effects of fenretinide.

## Results

### Sp1 and Brg-1 bind to the SPARC promoter in mammary carcinoma cell lines

**Figure 1:**
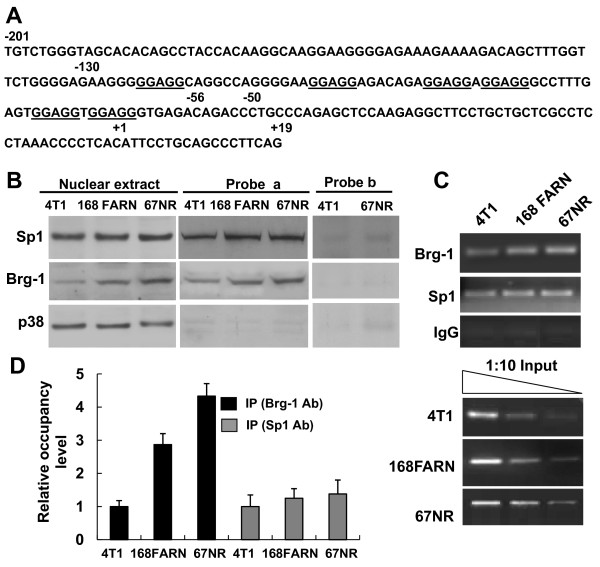
**Sp1 and Brg-1 are bound to the SPARC promoter in tumor cell lines**. (A) Nucleotide sequence (from - 201 to +19) indicating the position of six GGAGG boxes (underlined) in the mouse SPARC promoter. (B) Nuclear extracts were prepared from 4T1, 168FARN and 67NR cells. Proteins which bind to probe *a *(spanning GGAGG-rich nucleotides -130/-56) or probe *b *(spanning nucleotides -50/+19) were isolated from the nuclear extracts using the immobilized-template assay and then subjected to Western blot analysis with antibodies against Brg-1, Sp1 or p38. p38 and the probe *b *were used to serve as a non-specific binding control (right panel). Unpurified total nuclear extracts were also subjected to Western blot analysis and probed with the same antibodies (left panel). (C) Cross-linked and sonicated chromatin samples were prepared from 4T1, 168FARN and 67NR cells. ChIP assays were performed using antibodies against Sp1, Brg-1 or non-specific rabbit IgG as a control. Immunoprecipitated DNA and serially diluted input genomic DNA was amplified with primers specific to the SPARC promoter. PCR products were then analyzed using 2.0% agarose gels and stained with ethidium bromide. The illustrated results are representative of three independent experiments. (D) The ChIP DNA and input genomic DNA from (C) were quantified and amplified by real-time qPCR as described in the Materials and Methods section. The occupancy level of Brg-1 or Sp1 at the SPARC promoter is represented as the ratio of signal from IP samples versus that of the input minus background of IgG control. The relative occupancy level of Brg-1 or Sp1 in 4T1 cells is set as 1. Data represented as mean ± SEM (n = 3).

The 5'-flanking region of the mouse SPARC gene has a single major transcriptional start site; no TATA or CAAT boxes but six repeats of a GGAGG sequence within the proximal region of the SPARC promoter (-124/+19) (Figure 1A), as described previously [52]. These characteristics are well conserved in the SPARC gene among various species, including mouse, chicken, cow and human [53]. This GGAGG sequence represents one type of the regulatory sequence GC box (GC-I), which has been shown to bind transcription factor Sp1/3, regulating transcriptional activity of several genes including Adam8, Pgr and Adamts1 [54-56].

To determine whether Sp1 and Brg-1 bind to the GGAGG repeats sequence present in the mouse SPARC promoter, we employed an immobilized-template assay using a biotin-tagged probe *a *(spanning from nucleotide -130 to -56 of the SPARC promoter, Figure 1A), which allowed us to isolate the transcription factors that bind to this probe. Using Western blot analysis, we were able to document that both Sp1 and Brg-1 interacted with this region of the SPARC gene (Figure 1B, middle panel). The negative control, p38, was not associated with the probe *a*. A probe *b *(spanning from nucleotide -50 to +19 of the SPARC promoter), used as a non-specific binding control, was bound neither by Sp1 nor by Brg-1 (Figure 1B, right panel). The three well characterized mammary tumor cell lines (4T1, 168FARN and 67NR) used in our study are derived from a single, spontaneously arising mouse mammary tumor which differ from each other in their metastatic potential [57]. When injected into the mammary gland of mice, these tumor lines are either non-metastatic (67NR), spontaneously metastatic to lymph node (168FARN) or metastatic to lung, liver, bone and brain via the hematogenous route (4T1) [58]. The nuclear expression of Sp1 and Brg-1 were also determined by Western blot analysis. As shown in Figure 1B (left panel), there was no obvious difference in the protein level of Sp1 present in the three cell lines; whereas the protein level of Brg-1 declined in a concordant manner with the increase of metastatic potential, the latter being highest in non-metastatic 67NR cells, intermediate in partly metastatic 168FARN cells and lowest in highly metastatic 4T1 cells. To investigate whether Sp1 and Brg-1 are associated with the proximal region of the SPARC gene in living cells, we performed ChIP assays using antibodies to Sp1 and Brg-1, and nonspecific IgG as a control. A pair of primers spanning a 179-bp DNA fragment (-201/-23) of the proximal region encompassing the GGAGG repeats was used for PCR. As shown in Figure 1C, both the antibodies recognizing Sp1 and Brg-1 precipitated this promoter fragment, while the nonspecific IgG failed to do so. Further, the occupancy levels of Brg-1 and Sp1 at the SPARC promoter were analyzed by quantitative ChIP analysis. We found that, consistent with the nuclear Brg-1 protein level, Brg-1 occupancy likewise decreased in a concordant manner with the increase of metastatic potential in the three cell lines. The difference of Sp1 occupancy level was not significantly different among the three cell lines (Figure 1D). Our results demonstrate that both Sp1 and Brg-1 bind to the same proximal region of the SPARC promoter and that the level of Brg-1 expression and its binding to the SPARC promoter seem to negatively correlate with the metastatic potential of the three cell lines.

### Sp1 is essential for the recruitment of Brg-1 to the SPARC promoter

**Figure 2:**
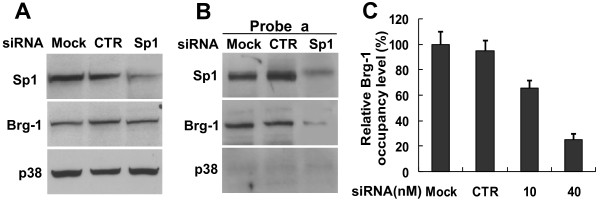
**Sp1 mediates the recruitment of Brg-1 to the proximal region of the SPARC gene**. 4T1 cells were transfected with mock (transfection reagent only), siRNA control (CTR), or siRNA specific for Sp1, and then cultured for 72 hrs prior to harvesting the cells. (A) Total cell extracts were prepared from transfected cells, and protein expression was analyzed by Western blotting using antibodies specific to Sp1, Brg-1 or p38. (B) Nuclear extracts were prepared and the binding reactions of Sp1, Brg-1 or p38 to the probe *a *were analyzed using the immobilized-template assay followed by Western blot analysis. (C) Cells were cross-linked and sonicated, and ChIP assay was performed. The precipitated DNAs and input DNA were quantified and then amplified using real-time qPCR. The occupancy level of Brg-1 at the SPARC promoter is calculated as described in Figure 1 D. The relative occupancy level of Brg-1 in mock-treated cells is set as 100%. Data represented as mean ± SEM (n = 3).

To determine whether Sp1 is specifically responsible for recruitment of Brg-1 to the SPARC promoter, we knocked down Sp1 expression using RNA interference. The effect of Sp1 knockdown on the binding of Brg-1 to the GGAGG repeats sequence of the SPARC promoter was then analyzed by using an immobilized-template assay. As shown in Figure 2A, transfection of specific Sp1 siRNAs into 4T1 cells led to a substantial down-regulation of Sp1 protein levels (decreased by > 76%), without any effect on Brg-1 protein expression. Transfection using a control siRNA had no effect on either Sp1 or Brg-1 levels. Interestingly, binding of Brg-1 to the SPARC promoter was significantly diminished when using nuclear extract from Sp1 siRNA-transfected cells (Figure 2B). To further test the role of Sp1 in mediating Brg-1 binding to the SPARC promoter in living cells, a ChIP-qPCR assay was performed using 4T1 cells transfected with Sp1 siRNA. As shown in Figure 2C, transfection with Sp1 siRNA significantly diminished the amount of Brg-1 bound to the SPARC promoter. These results suggest that the Sp1 transcription factor is essential for efficient binding of Brg-1 to the SPARC promoter.

### Interaction of Sp1 and Brg-1 in intact cells

**Figure 3:**
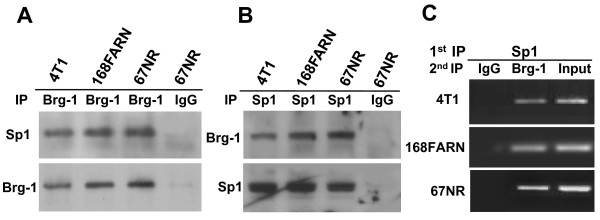
**Brg-1 and Sp1 are components of the same nuclear complexes**. (A) Nuclear extracts from 4T1, 168FARN and 67NR cells were immunoprecipitated (IP) using Brg-1 antibodies or rabbit IgG (control). The immunoprecipitates were subjected to SDS/PAGE gel followed by Western blot analysis using antibodies against Sp1 and Brg-1. (B) The co-immunoprecipitation assay was carried out by using Sp1 antibody or rabbit IgG for the IP and Brg-1 or SP1 antibodies for the Western blot analysis. (C) 4T1, 168FARN and 67NR cells were cross-linked and subjected to sonication and immunoprecipitation with the indicated antibodies. ChIP was first carried out using the Sp1 antibody, and the immunocomplexes were eluted using 10 mM dithiothreitol. The aliquots of the diluted elution were immunoprecipitated with Brg-1, or non- specific IgG as a control. The precipitated DNA fragments were amplified by PCR using the primers specific to the SPARC promoter region containing GGAGG repeats (spanning nucleotides -201/-23). The illustrated results are representative of three independent experiments.

Since depletion of Sp1 diminished Brg1 binding to the SPARC promoter, we postulated that Brg-1 is recruited to the SPARC promoter by forming a complex with Sp1. To test this hypothesis, we performed co-immunoprecipitation experiments using nuclear extracts from 4T1, 168FARN and 67NR cells. As shown in Figure 3A, Sp1 was found in the complex with Brg-1, but not with the IgG control. These co-immunoprecipitation assays were repeated in a reciprocal fashion using an anti-Sp1 antibody. Association of Sp1 and Brg-1 was again detected by immunoblotting of these complexes with anti-Brg-1 antibodies (Figure 3B). We next determined by re-ChIP analyses whether Brg-1 is associated with the Sp1-containing SPARC promoter fragment in living cells. Briefly, chromatin fragments from cross-linked 4T1, 168FARN and 67NR cells were first immunoprecipitated using antibodies against Sp1. The resulting immunocomplex was then eluted and subjected to immunoprecipitation using antibodies against Brg-1. PCR analysis was then used to determine whether the promoter fragment was present in the final precipitate. As shown in Figure 3C, we found that the promoter fragment present in the first immunocomplex generated using anti-Sp1 antibodies was pulled down again by anti- Brg-1 antibodies. These results indicate an association between Brg-1 and Sp-1 at the proximal promoter region containing GGAGG repeats in these cells.

### Brg-1 is required for expression of the SPARC gene

**Figure 4:**
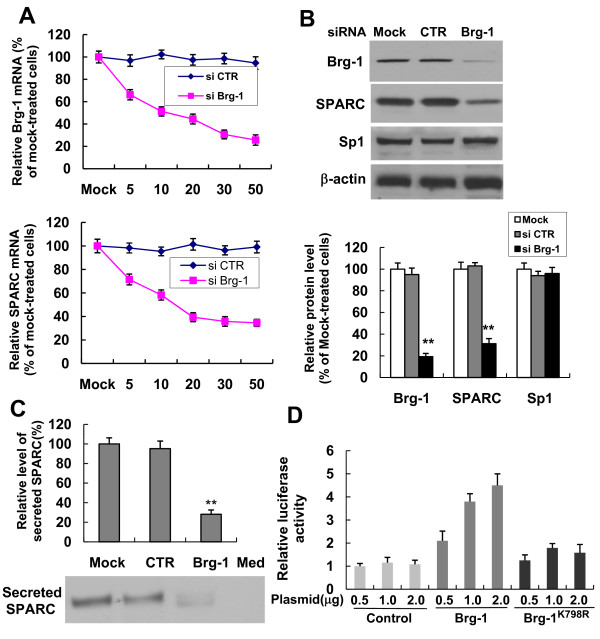
**Brg-1 is required for SPARC gene expression**. RNA interference- mediated depletion of Brg-1 reduces SPARC expression. 4T1 cells were transfected with mock, siRNA control or different concentrations of siRNA targeting Brg-1[0(mock)-50 nM] and were then cultured for another 48 hrs prior to harvesting the cells. (A) Total RNA was isolated from transfected 4T1 cells and mRNA levels of Brg-1 and SPARC were analyzed using real-time RT-qPCR. The relative mRNA level of Brg-1 or SPARC was represented as a percentage of the Brg-1 or SPARC mRNA level in mock-treated 4T1 cells (data shown are mean ± SEM, n = 4). (B) Total cell extracts were prepared from siRNA-transfected (30 nM) or mock-transfected 4T1 cells. Western blot analysis was performed to assess the protein expression levels of Brg-1, SPARC, Sp1 and β-actin (upper panel). Quantification of the protein expression of Brg-1, SPARC and Sp1 (data shown are mean ± SEM, n = 3) (bottom panel). **P < 0.001, compared with mock-treated cells. (C) Conditioned media were collected from siRNA-transfected (30 nM) or mock-transfected 4T1 cells, and the levels of secreted SPARC were assessed by Western blot analysis. Cell-free medium (Med) was used as a control. Densitometric quantitation was performed and results were adjusted for total protein content of cell lysate (densitometry/μg cell total protein). The relative level of secreted SPARC in the medium of mock-transfected 4T1 cells is set as 100%. Data represented as mean ± SEM (n = 3). (D) Overexpression of Brg-1 enhances the SPARC promoter-driven reporter gene transcription. 4T1 cells were co-transfected with the luciferase reporter vector pREP4-SP-Luc and expression plasmids encoding Brg-1 or its mutant (Brg-1^K798R^) or empty plasmids (control). Cell lysates were prepared 48 hrs after transfection and assayed for luciferase activity. Luciferase relative activity is expressed as a fold of the luciferase activity of the cells untransfected with Brg-1. The data shown (mean ± SEM) are the averages of three independent experiments performed in triplicate.

To further confirm the importance of Brg-1 in SPARC gene expression, we used RNA interference to deplete endogenous Brg-1. As shown in Figure 4A, transfection of 4T1 cells with different concentrations of siRNA targeting Brg-1 (0-50nM) resulted in an inhibition of Brg-1 mRNA expression in a dose-dependent manner (upper panel), accompanied by a dose-dependent decrease in SPARC mRNA level (bottom panel). Furthermore, Western blot analysis showed that transfection with 30 nM of Brg-1 siRNA led to a substantial down-regulation of Brg-1 protein levels (>80% decrease), with no effect on transcription factor Sp1 and β-actin expression. Transfection using a control siRNA had no effect on Brg-1, Sp1 or β-actin protein levels. Similarly to what was observed in the Sp1 knockdown experiments also, SPARC protein level was significantly inhibited in Brg-1 knockdown cells (reduced by 69%, Figure 4B). As SPARC is a secreted protein, in order to investigate if Brg-1 knockdown also leads to a decrease of SPARC secretion into the extracellular medium, cell media were collected for Western blot analysis. As shown in Figure 4C, a marked decrease in secreted SPARC was detected in the media harvested from Brg-1 siRNA-transfected cells in comparison to mock-treated cells. The cell-free medium (Med) was used as a control and no signal was detectable using the specific antibody against murine SPARC. To correct for any potential discrepancy in cell number resulting from different experimental conditions, densitometric quantitation was performed and results were adjusted for total protein amounts of cell lysates. After correction, SPARC secretion reduced by 72% in Brg-1 knockdown cells compared with mock-treated cells (*P *< 0.001). To further study the influence of Brg-1 on SPARC promoter activity, we generated a reporter pREP4-SP-Luc which contains a 220 bp fragment of the SPARC promoter (spanning nucleotides -201 to +19). This particular promoter region of the SPARC gene was previously shown to be necessary and sufficient to maintain constitutive SPARC gene expression, and our present results demonstrated that both Sp1 and Brg-1 are bound to this promoter region. The SPARC promoter-luciferase reporter was then introduced into 4T1 cells with an empty plasmid (as a control) or a plasmid expressing wild-type Brg-1 or mutant Brg-1 (K798R) defective in ATPase activity. Luciferase activity was determined 48 hrs later. As shown in Figure 4D, overexpression of wild-type Brg-1 significantly augmented the luciferase activity driven by the SPARC promoter, whereas mutant Brg-1 (K798R) had a much less pronounced enhancing effect on the luciferase activity. These results document a causal relationship between the level of Brg-1 protein and SPARC gene expression.

### Brg-1 enhances SPARC promoter activity in a Sp1-dependent manner

**Figure 5:**
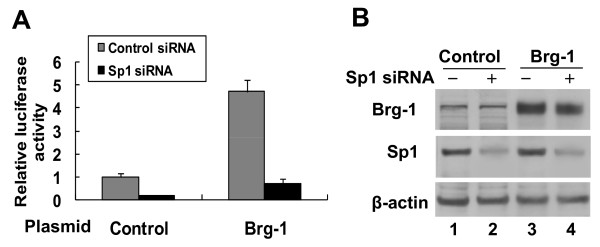
**Inhibition of endogenous Sp1 protein decreased the Brg-1-mediated transcriptional activation of the SPARC gene**. 4T1 cells were respectively transfected with siRNA control (-) or siRNAs specific for Sp1 (+). 24 hrs after transfection, the luciferase reporter construct pREP4-SP-luc was co-transfected with Brg-1 expression plasmid or empty plasmid (control) into 4T1 cells. Cell lysates were prepared 48 hrs after the second transfection. (A) The luciferase activity was analyzed. The data shown (mean ± SEM) are the averages of three independent experiments performed in triplicate. (B) Protein expression levels of Sp1, Brg-1 and β-actin (internal control) in transfected 4T1 cells were assessed using Western blot analysis. The illustrated result is a representative of three independent experiments.

To test if the Sp1 is required for the Brg-1-mediated transcriptional activation of the SPARC gene, we introduced Sp1 siRNAs and Brg-1 expression plasmids into the 4T1 cells to inhibit the expression of the Sp1 and enhance the expression of the Brg-1. As shown in Figure 5A, overexpression of Brg-1 increased the SPARC promoter-driven reporter gene expression by 4.7-fold in 4T1 cells without Sp1 knockdown, whereas knocking down Sp1 reduced the induction of SPARC promoter-driven reporter gene expression by Brg-1 from 4.7-fold to 0.7-fold. These results demonstrate that Sp1 plays an important role in Brg-1 mediated transcriptional activation of the SPARC gene.

To exclude the possibility that Sp1 siRNAs may modify Brg-1 protein expression, we examined Brg-1 protein levels in 4T1 cells transfected with control siRNA or siRNA specific for Sp1. As shown in Figure 5B, knockdown of Sp1 did not make a significant impact on the Brg-1 expression level (compare lanes 1 and 2 as well as lanes 3 and 4). The effect of Brg-1 overexpression on the Sp1 protein expression was also analyzed in 4T1 cells transfected with empty or Brg-1-encoding plasmids. Similarly, Sp1 protein expression was not affected significantly by the overexpression of Brg-1 protein (compare lanes 1 and 3).

### Endogenous SPARC expression correlates with Brg-1 expression levels

**Figure 6:**
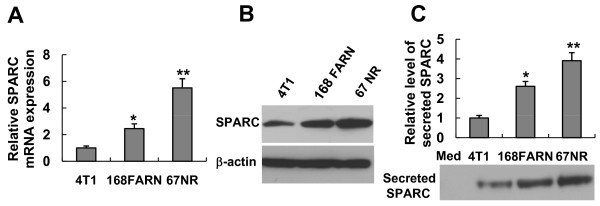
**Analyses of SPARC expression and secretion from mammary tumor cell lines with different metastatic capacities**. Cells were seeded into 6-well plates at a density of 1 × 10^5 ^cells (4T1 or 168FARN) or 1.5 × 10^5 ^cells (67NR) per well and cultured at 37°C. Cell-free medium served as a control. Seventy-two hrs after culture, cell media were collected, and total RNA as well as protein extracts were prepared from the three cell lines. (A) Real-time qRT-PCR was used to analyze the relative expression of SPARC mRNA level. The SPARC mRNA was normalized by GAPDH expression and the relative expression level is represented as a fold of the SPARC mRNA level in 4T1 cells. (Data represented as mean ± SEM, n = 4). *P < 0.01, compared with 4T1; **P < 0.01, compared with 168FARN. (B) Cellular SPARC protein expression was assessed using Western blot analysis. The illustrated result is a representative of three independent experiments. (C) The levels of secreted SPARC protein in the culture media were assessed by Western blot analysis. Cell-free medium (Med) was used as a control. Densitometric quantitation was performed and results were adjusted for total protein content of cell lysate (densitometry/μg cell total protein). The relative level of secreted SPARC in the medium of 4T1 cells is set as 1. Data represented as mean ± SEM (n = 3). *P < 0.01, compared with 4T1; **P < 0.01, compared with 168FARN.

We found that the endogenous expression of Brg-1 decreased in a concordant manner with the increase of metastatic potential among the three tumor cell lines. We next documented a causal relationship between the level of Brg-1 protein and SPARC gene expression in 4T1 cells. These observations led us to believe that the difference of Brg-1 gene expression could be responsible for the differential expression of SPARC in the three cell lines. To investigate, the SPARC mRNA and protein levels were assessed. As we expected, in parallel with the Brg-1 protein level, the SPARC mRNA and protein levels were highest in 67NR, intermediate in 168 FARN and lowest in 4T1 cells (Figures 6A, B and Figure 1B, left panel). In addition, Western blot analysis of secreted proteins demonstrated that the amount of released SPARC in the conditioned medium is correlated with the intracellular mRNA and protein expression levels of this gene (compare Figure 6C with Figure 6A and 6B). These results suggest that the SPARC expression and secretion are correlated with the Brg-1 expression level. Our results also suggest that higher expression and secretion of SPARC might be correlated with lower metastatic potential of mammary carcinoma cells.

### Expression and secretion of the SPARC is up-regulated by fenretinide treatment in mammary tumor cell lines

**Figure 7:**
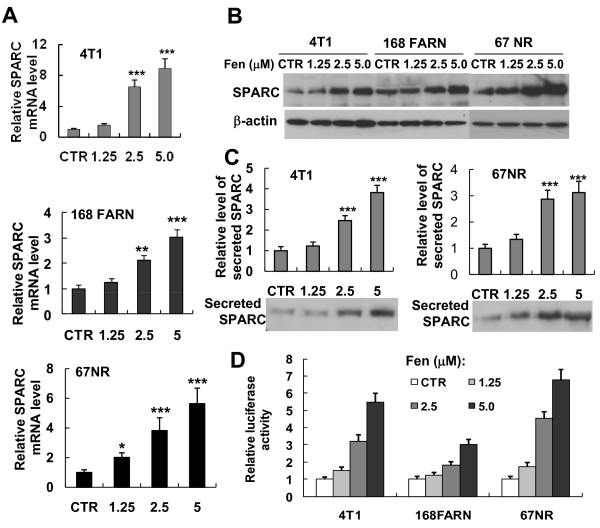
**Fenretinide treatment increases SPARC expression and secretion from mammary carcinoma cells**. 4T1, 168FARN or 67NR cells were treated with various concentrations of fenretinide (1.25 μM, 2.5 μM and 5 μM) or left untreated (control) for 24 hrs. (A) Total RNA was isolated from different cell lines and SPARC mRNA level was analyzed using real-time RT-qPCR. Experiments were conducted in quadruplicate and normalized to GAPDH mRNA level. The relative SPARC mRNA level is represented as a fold of the SPARC mRNA level in untreated cells (data shown are mean ± SEM, n = 4). Compared with control, *P < 0.05, **P < 0.01, ***P < 0.001. (B) Total protein extracts were prepared from different cell lines and expression of SPARC protein levels were assessed using Western blot analysis. Expression of β-actin was used as an internal control. (C) Conditioned media were collected from 4T1 (left panel) and 67NR (right panel) cell lines and the levels of secreted SPARC protein were assessed by Western blot analysis. Densitometric quantitation was performed and results were adjusted for total protein content of cell lysate (densitometry/μg cell total protein). The relative level of secreted SPARC in the medium of untreated cells is set as 1. Data represented as mean ± SEM (n = 3). Compared with control, ***P < 0.001. (D) Effect of fenretinide treatment on the transcriptional activity of the SPARC promoter. 4T1, 168FARN and 67NR cells were transiently co-transfected with the luciferase reporter vector pREP4-SP-Luc and pRL-CMV. The pRL-CMV reporter was used as an internal control. 24 hrs after transfection of plasmids, cells were cultured for 24 hrs with or without different concentrations of fenretinide, and the luciferase activity was analyzed by the dual-luciferase reporter assay system. Luciferase relative activity is presented as a fold of the luciferase activity of the cells without fenretinide treatment. The data shown (mean ± SEM) represent the averages of three independent experiments performed in triplicate.

Fenretinide has been shown to have a long-term protective effect against secondary breast cancers [45,51,59]. Since we observed an interesting association between the relatively low level of SPARC expression and the high metastatic potential of 4T1 cells, we postulated that treatment with fenretinide might reverse the tumorigenic ability of these cells by affecting the expression of SPARC. To test this hypothesis, the expression of SPARC was detected by RT-qPCR and Western blot analysis in 4T1, 168 FARN and 67NR cells treated with fenretinide at different concentrations (0-5 μM) for 24 hrs. As shown in Figures 7A and B, fenretinide induced the expression of SPARC mRNA and protein in a dose-dependent manner. To investigate if fenretinide affects the secretion of SPARC protein, culture media were collected from the 4T1 and 67NR cells treated with fenretinide for 24 hrs and secreted SPARC was assessed. As expected, fenretinide increased the secretion level of SPARC into the extracellular media in a dose-dependent manner, too (Figure 7C). To test whether the fenretinide-induced expression of SPARC resulted from the transcriptional activation, reporter vectors (pREP4-SP-Luc) were transfected into the three cell lines. The transfected cells were then left untreated or treated with different concentrations of fenretinide for 24 hrs. As shown in Figure 7D, fenretinide increased SPARC promoter-driven luciferase activity in all three cell lines in a dose-dependent manner. The results demonstrate that fenretinide treatment increases the transcriptional activity and expression of the SPARC gene.

### Effects of Sp1/Brg-1 on the fenretinide-induced transactivation of SPARC gene

**Figure 8:**
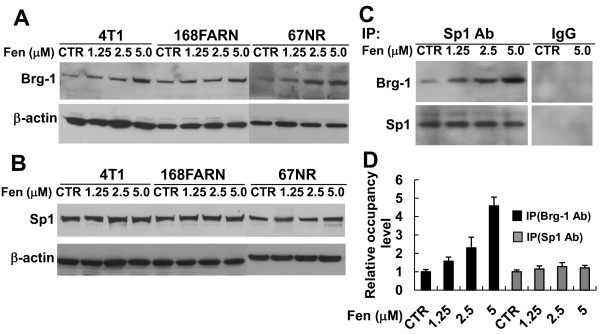
**Fenretinide induces the expression of Brg-1, Brg-1 and Sp1 complex formation and the binding of Brg-1 to SPARC promoter**. 4T1, 168FARN or 67NR cells were left untreated (control) or treated with the indicated doses of fenretinide for 24 hrs. Cell lysates were prepared, and assessed by Western blot analysis for Brg-1 (A) and Sp1 (B), respectively. Expression of β-actin was detected as an internal control. (C) Nuclear extracts from fenretinide-treated 4T1 cells were immunoprecipitated (IP) with antibodies against Sp1 or IgG (control). The precipitated protein complexes were subjected to SDS/PAGE and analyzed by Western blot analysis with an antibody against Brg-1 or Sp1. (D) Cross-linked chromatin derived from either fenretinide-treated or untreated 4T1 cells was immunoprecipitated with Brg-1or Sp1 antibodies or nonspecific IgG. The precipitated DNAs or input DNA were analyzed by real-time qPCR with the specific primers for the region from -201 to -23 nucleotides of the SPARC promoter. The relative occupancy level of Brg-1 or Sp1 in untreated 4T1 cells (CTR) was set as 1. Data shown are mean ± SEM (n = 3)

To explore in detail fenretinide-induced transcriptional activation of the SPARC gene, the expression and the ability of Brg-1 and Sp1 binding to the SPARC promoter were analyzed in all three cell lines in response to fenretinide treatment. As shown in Figures 8A and 8B, fenretinide induced the expression of Brg-1 in a dose-dependent manner, while it had no significant effect on the expression of Sp1. In addition, treatment of cells with fenretinide increased the interaction between Sp1 and Brg-1 (Figure 8C). To directly examine the effect of fenretinide on the binding of Brg-1 and Sp1 proteins to the SPARC promoter, we performed ChIP-qPCR to assess the occupancy level of Brg-1 and Sp1. As shown in Figure 8D, the occupancies of Brg-1 protein at the selected promoter region increased upon fenretinide treatment in a dose-dependent manner. Fenretinide had slight/no effect on occupancy level of Sp1 at this region. These results suggested that fenretinide induced the formation of the Sp1/Brg-1 complex, resulting in facilitating the access of Brg-1 to the SPARC promoter and enhancing the transactivation of this gene.

### Brg-1 mediates the fenretinide-induced transactivation of SPARC

**Figure 9:**
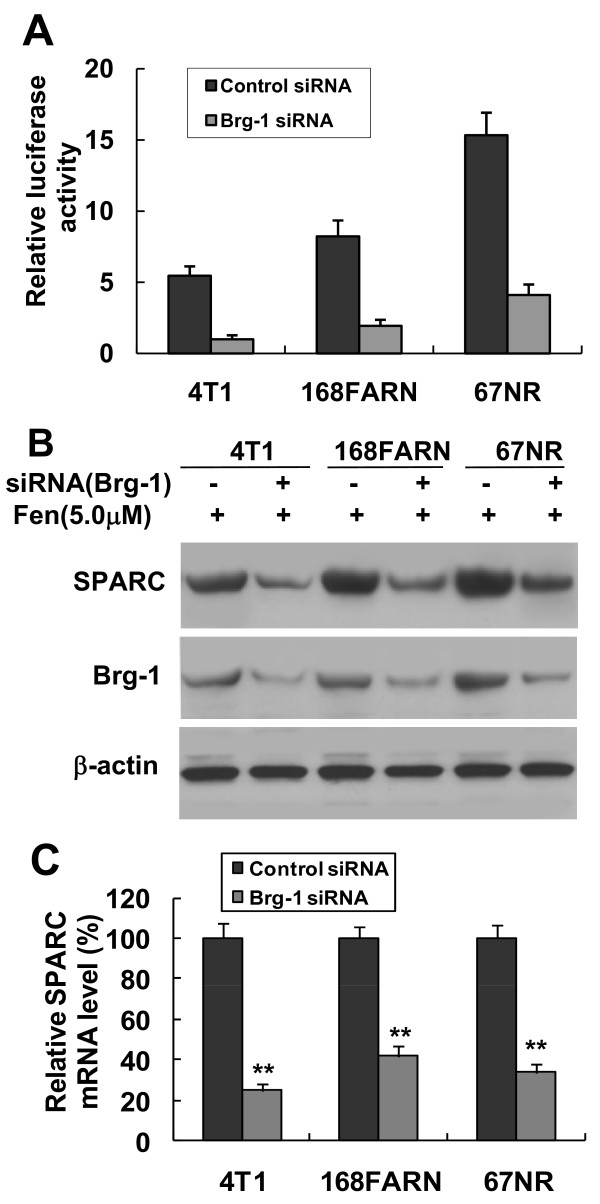
**Fenretinide-induced transactivation and expression of SPARC depend on Brg-1**. 4T1, 168FARN or 67NR cells were respectively transfected with siRNA control (-) or siRNA specific for Brg-1 (+). 24 hrs after transfection, cells were transiently co-transfected again with the luciferase reporter vector pREP4-*SP*-Luc and pRL-CMV. 6 hrs after the second transfection, cells were cultured for 24 hrs with or without fenretinide (5 μM). Total protein extracts were prepared. (A) The luciferase activities were analyzed by the dual-luciferase reporter assay system. (B) Expression of SPARC and Brg-1 proteins were analyzed using Western blot analysis. β-actin was analyzed as a control. (C) Cells were transfected with Brg-1 siRNA or control siRNA for 24 hrs and then treated with fenretinide for 24 hrs. Total RNA was extracted and expression of SPARC mRNA levels was measured by real-time RT-qPCR. Experiments were conducted in quadruplicate and normalized to GAPDH mRNA level. The relative SPARC mRNA level in control siRNA-treated cells is set as 100% (data shown are mean ± SEM, n = 4, **P < 0.001)

Since the expression of Brg-1 as well as the formation and binding of Sp1/Brg-1 to the SPARC promoter were increased in fenretinide-treated cells, it is likely that Brg-1 is involved in fenretinide-induced transcriptional activation of the SPARC gene. We employed Brg-1 siRNAs to knockdown Brg-1, and its effect on fenretinide-induced transcriptional regulation of the SPARC gene was then assessed by luciferase reporter assays. As shown in Figure 9A, fenretinide-induced promoter activity was significantly inhibited in Brg-1 knockdown cells. Interestingly, fenretinide-induced SPARC gene expression was significantly inhibited both at the protein and mRNA levels in Brg-1 knockdown cells (Figures 9B and 9C, respectively). These results reveal that the Sp1/Brg-1 complex plays an important role in fenretinide-induced SPARC gene expression, demonstrating that the Brg-1 protein is responsible for fenretinide-induced SPARC gene transactivation.

### SPARC is involved in fenretinide-induced cell invasion inhibition

**Figure 10:**
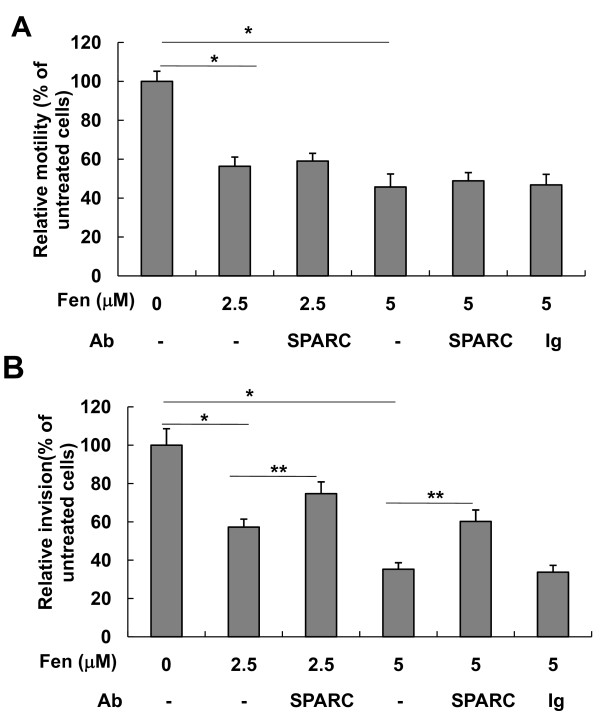
**SPARC is a mediator for fenretinide inhibiting cell invasion but not cell motility**. (A) Cell motility was analyzed by *in vitro *wound assay. 4T1 cells were grown on 24-well culture plates overnight and were then left untreated or pretreated with 2.5 μM or 5.0 μM fenretinide for 6 hrs. The confluent cell monolayers were gently scratched with a pipette tip to produce a wound. After wash, the cells were cultured in medium containing different combinations of fenretinide with anti-SPARC antibodies. Quantitative analysis was performed 18 hrs later, as described in the Materials and Methods section. Data shown are means ± SEM. *P < 0.01. (B) *In vitro *invasion assay was performed using 24-well trans-well units with polycarbonate filters (pore size 8 μm) coated on the upper side with ECMatrix™. Cells pretreated or untreated with fenretinide for 6 hrs were collected, and 5 × 10^4 ^cells in 0.3 ml of serum-free medium with different combinations of fenretinide with anti-SPARC antibodies were placed in the upper part of the trans-well unit and allowed to invade for 24 hrs. The lower chamber of the plate was filled with medium containing 10% FBS. The relative invasion of cells cultured in medium without fenretinide and anti-SPARC antibodies was considered as 100%. Results from three independent experiments were expressed as mean ± SEM. *P < 0.01, compared with untreated cells. **P < 0.01, compared with cells treated with fenretinide alone.

Fenretinide has been shown to inhibit the invasion of several cancer cell types, including breast cancer [60], ovarian cancer [61], prostate cancer [62] and Kaposi's sarcoma [63]. Therefore, we next tested whether fenretinide as well as fenretinide-induced SPARC expression have effects on the migration and invasion of 4T1 cells. Our results demonstrated that fenretinide suppressed the cell motility in a dose-dependent manner and the inhibition of cell motility was not significantly affected by addition of anti-SPARC antibodies (Figure 10A). Similarly, 4T1 cells treated with fenretinide showed a significant decrease in cell invasion compared with untreated cells. However, when cells were incubated with anti-SPARC antibodies, we found that cell invasion inhibited by fenretinide treatment was partly restored. On the contrary, addition of non-specific goat IgG had no effect (Figure 10B). The results suggest that SPARC is one of the mediators involved in the fenretinide-induced suppression of cell invasion.

## Discussion

SPARC is a single-copy gene with a high degree of evolutionary conservation. The mouse SPARC gene is 92% identical to the human homologue. The 5'-proximal flanking region of the SPARC gene displays a well-conserved and characterized GGAGG repeats sequence [52,53]. The activity of the human SPARC promoter requires a purine-rich region with GGAGG repeats (within the -120/-70 fragment) in human breast cancer MCF7 cell line, and the transactivation of the SPARC promoter is dependent on the transcription factor Sp1/3 in Drosophila SL2 cells [64]. Furthermore, it was shown that Sp1/3 is required for constitutive activation of the chicken SPARC promoter (-124/+16), by directly binding to the GGA-rich, -92/-57 fragment [41]. These results suggest that constitutive transcription of SPARC might be regulated by similar mechanisms in various species. Indeed, our present study demonstrated that Sp1 is bound to the GGAGG repeat region within the mouse SPARC promoter, which is consistent with previous findings in chicken and human SPARC genes. What is more, we found for the first time that Sp1 is essential for the recruitment of Brg-1 to the SPARC promoter (within the -130/-56 fragment) via interaction with each other. We demonstrated that inhibition of Brg-1 significantly reduces the expression of the SPARC gene, and Brg-1 cooperates with Sp1 to enhance the SPARC promoter activity. These results suggest that Sp1 and Brg-1 work together to maintain a constitutive expression level of the SPARC gene. We found that there exist significant differences in the levels of endogenous SPARC mRNA and protein expression as well as secreted SPARC among the three tumor cell lines, with these levels being highest in the non-metastatic 67NR cells, intermediate in the partly metastatic 168 FARN cells and lowest in the highly metastatic 4T1 cells. This could be explained, at least in part, by different expressions of Brg-1 as well as different Brg-1 binding levels at the SPARC promoter among the three cell lines.

Fenretinide has been shown to induce apoptosis leading to inhibition of mammary carcinogenesis [46,47]; it has also been shown to reduce the occurrence of secondary breast cancer in women aged 40 years or younger [45]. Our results demonstrated that fenretinide has the ability to induce the expression of Brg-1, which can explain one of the possible mechanisms responsible for the chemopreventive potential of this drug. It has been reported that a variety of human malignancies are associated with mutations of Brg-1, thus suggesting that Brg-1 may play an important role in tumor suppression [65]. Brg-1 has been shown to interact with the retinoblastoma tumor suppressor gene product (pRB), and induce cell cycle arrest through the repression of E2F target genes such as cyclin E, cyclin A, and CDC2 [66-68]. In addition, Brg-1 is also required for p53-and BRCA-1-mediated transcriptional activation [32], as well as tumor suppressor prohibitin- and HIC1-mediated transcriptional repression. Brg-1 heterozygous mice display higher susceptibility to mammary tumors [69], while complete loss of Brg-1 enhances lung cancer development [70]. Furthermore, Brg-1 has been demonstrated to be silenced or mutated in various human tumor cell lines derived from breast, ovarian, lung, brain and colon cancers [71], and the loss of Brg-1 expression is associated with a poor prognosis in lung cancer patients [72]. Another study showed that Brg-1 expression is also lost in 70.6% of established neuroendocrine carcinomas of the cervix [73]. The findings that Brg-1 is frequent lost in primary and metastatic melanomas and it interacts with the melanoma-associated tumor suppressor p16^INK4a ^imply an important role for Brg-1 in melanoma [74]. All these data suggest that Brg-1 may function as a tumor suppressor. Therefore, the anti-cancer effects of fenretinide might be partly due to its ability to induce Brg-1 expression. The induction of Brg-1 expression in response to fenretinide and its enhancing effect on apoptosis and tumor suppression are certainly worthy of more extensive studies.

Besides Brg-1, we also found that SPARC expression and secretion as well as SPARC promoter-driven transcriptional activity were induced by fenretinide in tumor cells. Moreover, our results revealed that knockdown of Brg-1 inhibited the fenretinide-induced SPARC expression and SPARC promoter-driven transcriptional activity. Together with these results, it is suggested that fenretinide up-regulates the SPARC transcription via the induction of Brg-1 expression. SPARC is an important regulator of cell growth and malignancy with complex biological effects that are cell- and tumor-type specific. For example, in certain types of cancers, such as melanomas and gliomas, SPARC is associated with a highly aggressive tumor phenotype, while in others, mainly in neuroblastomas, as well as in ovarian and colorectal cancers, SPARC functions as a tumor suppressor [18]. The role of SPARC in the development and progression of breast cancer is still not fully elucidated. Dhanesuan et al. [9] revealed that SPARC can inhibit breast cancer cell proliferation. A report using human MDA-MB231 breast cancer cells demonstrated that overexpression of SPARC inhibited the metastatic capacity of these cells to different organs, including lungs and bones [11]. Wong and colleagues recently reported that 60% of patients with low SPARC expression had metastases within 5 years of diagnosis, while only 33% of patients with high SPARC-expression developed metastasis in the same period [75]. Another recent study also revealed that down-regulation of SPARC is correlated with poor prognosis in breast cancer patients[76]. Therefore, these results seem to support the anti-tumorigenic role of SPARC. In this study, we detected the SPARC expression level and its secretion into milieu using three different mammary carcinoma cell lines with different levels of metastatic potential: highly metastatic 4T1, moderately metastatic 168 FARN and non-metastatic 67NR cell lines. The results showed that both SPARC gene expression and secretion levels were negatively associated with the metastatic capacity of tumor cells. We also found that the invasion activity in 4T1 cells treated with fenretinide was significantly decreased compared with untreated cells. Cancer cells treated with a SPARC antibody resulted in an abrogation of fenretinide-induced decrease in cell invasion. These results suggest that fenretinide is able to induce the expression of SPARC gene, and as a consequence, to inhibit cancer cell invasion.

## Conclusions

In summary, we found that Sp1/Brg-1 complex is involved in the constitutive expression of the SPARC gene in mammary tumor cells. We also demonstrated that fenretinide up-regulates SPARC transcription, expression and secretion via the induction of Brg-1 expression and that SPARC plays an important role in fenretinide-induced inhibition of cell invasion. Our findings provide new insights for the understanding of the anti-cancer effects of fenretinide on breast cancer.

## Materials and methods

### Cells and cell culture

Mammary tumor cell lines, 4T1, 168FARN and 67NR, were generously provided by Dr. F. Miller (Barbara Ann Karmanos Cancer Institute, MI, USA), and maintained in DMEM supplemented with 7% fetal bovine serum and 1% streptomycin-penicillin, and incubated in a humidified atmosphere containing 5% CO_2 _at 37°C.

### Plasmid constructs and reagents

pREP4-luc was constructed as described previously [77]. pREP-SP-luc was constructed by inserting the PCR-amplified SPARC promoter (spanning nucleotides -201/+19 of SPARC gene), using the forward primer (5'-GAGCTAGCTGTCTGGGT AGCACACAGCCTAC-3') and reverse primer (5'-CAAAGCTTCTGAAGGGCTGC AGGAATGTG-3'), into the *Nhe*I-*Hin*dIII sites of pREP4-luc. Expression plasmid encoding human Brg-1 (pBJ5 BRG1) and the ATPase-defective variant of Brg-1 (pBJ5 BRG1 DN, K798R mutant) [78] were obtained from AddGene (Cambridge, MA, USA), and human Brg-1 was shown to be expressed correctly and work properly in mouse cells and in frog oocytes [79,80]. Fenretinide (2,4,6,8-Nonatetraenamide, N-(4-hydroxyphenyl)-3,7-dimethyl-9-(2,6,6-trimethyl-1-cyclohexen-1-yl-(all-E); 374551) powder was generously provided by Dr. R. Smith (NIH, Bethesda, Maryland, USA).

### Western blot analysis

After appropriate treatments, cells were collected and total cellular extracts or nuclear fractions were prepared. Western blot analysis was then performed as described previously [81]. A monoclonal antibody against β-actin (Sigma, Saint Louis, MO, USA) was used at a 1:5,000 dilution. A monoclonal antibody against SPARC (R&D systems, Minneapolis, MN, USA) was reconstituted at a concentration of 500 μg/ml and used at a 1:2,500 dilution. A rabbit polyclonal antibody against Brg-1 (sc10768×, Santa Cruz Biotechnology, Santa Cruz, CA, USA) or Sp1 (sc14027×, Santa Cruz Biotechnology) was used at a 1:4,000 dilution.

### Immobilized-template assays

Immobilized-template assays were performed as described previously [82]. Two hundred micrograms of Dynabeads M280 streptavidin (Dynal) was prepared, concentrated and resuspended in 20 μl of buffer T (10 mM Tris [pH 7.5], 1 mM EDTA, 1 M NaCl) including 10 pmol of biotinylated GGAGG-rich-containing either probe *a *(spanning nucleotides -130/-56 of the *SPARC *gene [gene bank accession #M20683]) or probe *b *(spanning nucleotides -50/+19 of the *SPARC *gene). The mixture was gently agitated for 1 hr at room temperature (RT) and the beads were then washed 4 times in buffer T to remove unbound probes. Bead-coupled probes were equilibrated in buffer R (10 mM Tris [pH 7.5], 1 mM MgCl_2_, 0.1% NP-40, 1 mM EDTA, 10 mM DTT, 5% glycerol, 60 mM KCl, 12 mM HEPES [pH 7.9], 0.03% BSA) for 30 min, centrifuged and resuspended in buffer R containing 200 μg of nuclear extract and 40 ng/μl of poly (dG-dC) (120 μl final volume), and agitated for 30 min at RT. After binding reaction, the beads were washed three times using buffer R containing 10 ng/μl of poly (dG-dC). The bound proteins were eluted by boiling them in SDS sample buffer, and the presence of Brg-1, Sp1 and p38 were detected by Western blot analysis.

### Chromatin Immunoprecipitation (ChIP) assay, Re-ChIP and ChIP-qPCR

The ChIP assay was performed using a chromatin immunoprecipitation assay kit (Upstate, Lake Placid, NY) according to the manufacturer's instructions. Cells were fixed with 1% formaldehyde for 10 min, washed with ice-cold PBS containing protease inhibitors and lysed with SDS lysis buffer. The lysate was sonicated to yield DNA fragments between 300 and 1000 base pairs, and centrifuged at 13,000 rpm for 10 min. The supernatant was diluted and pre-cleared with salmon sperm DNA/protein A agarose. Immunoprecipitation was performed overnight at 4°C using either non-specific IgG or the antibodies against Brg-1 or Sp1. The immunoprecipitates were washed and eluted, and the cross-links were reversed. The precipitated DNA fragments were purified. The 5'-promoter region spanning nucleotide positions -201 to -23 from the transcription start site of the SPARC gene were amplified by PCR using 5'-TGTCTGGGTAGCACACAGCCTAC-3' and 5'-GCAGGAAGCCTCTT GGAGCTCT-3' primers. Re-ChIP assays utilized a similar protocol, except that the primary immunocomplex obtained with the Sp1 antibody was eluted by 10 mM dithiothreitol with agitation at 37°C for 30 min. The eluate was diluted 50 times with buffer (20 mM Tris-HCl, pH 8.1, 150 mM NaCl, 2 mM EDTA, and 1% Triton X-100) and immunoprecipitated using the second antibodies. For ChIP-qPCR assay, the precipitated DNA fragments were purified and quantified with the Quant-iT™ dsDNA Assay Kit (Molecular Probes, Eugene, Oregon) and were then amplified by real-time qPCR using the same primers as for regular PCR.

### Quantitative real-time PCR

Total RNA was extracted from cells using TRIzol Reagent (Invitrogen, Burlington, ON, Canada) according to the manufacturer's instructions. One μg of total RNA was reverse-transcribed with the QuantiTect reverse transcription kit (Qiagen, Mississauga, ON, Canada). An equal amount of cDNA or purified DNA fragment from ChIP was then amplified by real-time PCR using the Stratagene Mx-4000 and Brilliant SYBR Green QPCR Master Mix. Gene expression was normalized to a house-keeping gene (GAPDH) and the relative expression values between the samples were calculated based on the threshold cycle (C_T_) value using the 2^-ΔΔCT ^method [83]. The following primers were used for cDNA amplification: Brg-1, 5'-TCTGAGGTGGACGCCCGACACATTA-3' (forward) and 5'-TAAGGACCTGC GTCAACTTGCAGTG-3' (reverse); and SPARC, 5'-AGGTGTGTGAGCTGCACG AGA-3' (forward) and 5'-GAAGTGGCAGGA AGAGTCGAA-3'(reverse).

### Small RNA interference experiment

The transfection of siRNA into 4T1, 168FARN or 67NR cells was performed in 6-well plates using the Lipofectamine™ RNAiMAX (Invitrogen, Burlington, ON), according to the manufacturer's instructions. One day before transfection, cells were seeded at an appropriate density to give 40~50% confluence at the time of transfection. The siRNAs against Brg-1, Sp1 and control siRNA were purchased from Santa Cruz Biotechnology, Inc. (Santa Cruz, CA). Cells were harvested for assays 48 hrs or 72 hrs after transfection with these siRNAs. To assess luciferase activity, the cells were transfected 24 hrs after siRNA transfection with 1.8 μg of the luciferase reporter constructs and 100 ng of *Renilla *luciferase control vector (pRL-CMV). 24 hrs later, the cells were treated with fenretinide for another 24 hrs and luciferase activity was measured.

### Immunoprecipitation assays

Cells were washed once with ice-cold PBS and lysed in EBMK/0.1% NP-40 buffer (25 mM HEPES, pH 7.6, 5 mM MgCl_2_, 1.5 mM KCl, 75 mM NaCl, 175 mM sucrose, 0.1% NP-40 and protease inhibitors) on ice for 10 min. The nuclear pellet was collected by centrifugation at 500 × g for 4 min. and washed three times with EBMK buffer (no NP-40). The nuclei were then lysed in 1 ml of RIPA buffer containing protease inhibitors, passed repeatedly through a 22-gauge needle and centrifuged at 10,000 × g for 30 min. The supernatants were pre-cleared with protein A/G agarose for 30 min. Immunoprecipitation was performed overnight at 4°C using the antibody against Brg-1 or Sp1. To precipitate the antigen-antibody complex, protein A/G agarose was added and incubated for 1 hr at 4°C. After washing with RIPA buffer, the precipitated proteins were eluted by boiling in 2× SDS sample buffer and analyzed by immunoblotting using antibodies to Brg-1 or Sp1.

### Luciferase activity

Transient transfections of 4T1, 168FARN or 67NR cells were performed using the Lipofectamine™ 2000 and Plus reagent (Invitrogen, Burlington, ON) according to manufacturer's instructions. Briefly, cells were seeded into 12-well plates one day before transfection at a density of 5 × 10^4 ^cells (4T1 or 168FARN) or 1 × 10^5 ^cells (67NR) per well. Cells were transfected with 1.8 μg luciferase reporter constructs and 100 ng of *Renilla *luciferase control vector (pRL-CMV). 24 hrs after transfection, cells were treated with fenretinide or left untreated for 24 hrs before harvest. Luciferase reporter assays were performed using the Dual-Luciferase^® ^Reporter Assay System (Promega, Madison, WI) and the luminescence measurements were done with a Turner Designs model TD-20/20 luminometer. Firefly luciferase activity was normalized to Renilla luciferase activity. Each transfection was done in triplicate and repeated three times.

### Measuring secreted SPARC

Following the treatment of cells with siRNA or fenretinide, cell media were collected and centrifuged to remove cell debris. Equal volumes (15 μl) of supernatant were used for Western blot analysis. Densitometric quantitation was performed, and to correct for any potential discrepancy in cell number resulting from different experimental conditions, the densitometric results were adjusted for total protein contents of cell lysates.

### *In vitro *wound healing assay

Cells were seeded in 24-well culture plates at 1.5 × 10^5 ^cells/well. After 18 hrs, the cells were left untreated or pretreated with 2.5 μM or 5.0 μM fenretinide for 6 hrs before wound formation. The *in vitro *'scratch' wounds were created by scraping the confluent cell monolayer with a 200 μl pipette tip and cultures were then washed twice with PBS to remove floating cells. Cells were then cultured in fresh medium or medium containing 2.5 μM or 5.0 μM fenretinide alone or combined with 8 μg/ml goat nonspecific IgG or SPARC antibody (R&D systems). The plates were photographed at 0 hr and 18 hrs after treatment. The wound width was measured using the program Image J http://rsbweb.nih.gov/ij/ between two certain points on either side of the gap. For proper statistical evaluation, at least three measurements at different points were performed at each image. The wound width at the 18-hr time point was subtracted from that at the 0-hr time point. The distance was normalized to the wound width at 0 hr. The values were then expressed as relative motility, setting the cell motility of untreated cells as 100%. Three independent experiments were done in triplicates.

### Invasion assay

Cell invasion ability was assessed using a cell invasion assay kit (Chemicon International, Temecula, CA, USA) according to the manufacturer's instructions. The assay was performed in an invasion chamber, which consists of a 24-well tissue culture plate containing 12 cell culture inserts. The inserts contain an 8-μm pore size polycarbonate membrane coated on the upper side with a thin layer of ECMatrix™. Cells were pretreated with 2.5 μM or 5.0 μM fenretinide or left untreated for 6 hrs and were then collected and counted. 5 × 10^4 ^untreated cells were resuspended in 0.3 ml of serum-free medium and pretreated cells were resuspended in 0.3 ml of serum-free medium containing 2.5 μM or 5.0 μM fenretinide only or combined with goat nonspecific IgG (5 μg/ml) or SPARC antibody (5 μg/ml). The lower chamber of the plate was filled with 0.5 ml medium containing 10% FBS with or without fenretinide. The cell suspension was then placed in the upper chamber and incubated at 37°C for 24 hrs. The noninvasive cells on the upper side of the membrane were removed. The invasive cells on the lower surface of the membrane were stained and then lysed. Absorbance was measured with a microplate reader at 560 nm. Each experiment was repeated three times, and the data represent the mean ± SEM of three determinations.

### Statistical analysis

All data are presented as means ± SEM of three or four experiments. Analysis was performed using unpaired Student's *t *test. *P *< 0.05 was considered significant.

## Abbreviations

SPARC: Secreted protein, acidic and rich in cysteine; Brg-1: Brahma-related gene-1; MMP-2: matrix metalloproteinase-2; MMP-9: matrix metalloproteinase-9; TopBP1: DNA topoisomerase IIβ binding protein I; HIC1: hypermethylated in cancer 1; BRCA-1: breast cancer-1; ChIP: Chromatin immunoprecipitation; siRNA: small interfering RNA

## Competing interests

The authors declare that they have no competing interests.

## Authors' contributions

YZX designed the study, performed plasmid construction, ChIP, co-immunoprecipitation, cell transfection, luciferase reporter analysis, cell migration and invasion assays, data analysis as well as prepared the draft version of the manuscript. MH and TT performed cell culture, RNA expression analysis, Western blot analysis, statistical analysis and helped YZX in most experiments. SDM was involved in the design of the study, and specifically performed the immobilized-template assays. TM supervised the study and contributed to the manuscript preparation. DR designed and coordinated the study, and revised the manuscript. All authors have read and approved the final manuscript.
